# Has the Impact of Rising CO_2_ on Plants been Exaggerated by Meta-Analysis of Free Air CO_2_ Enrichment Studies?

**DOI:** 10.3389/fpls.2016.01153

**Published:** 2016-08-03

**Authors:** Matthew Haworth, Yasutomo Hoshika, Dilek Killi

**Affiliations:** ^1^Tree and Timber Institute, National Research Council (CNR-IVALSA), FlorenceItaly,; ^2^Institute for Sustainable Plant Protection, National Research CouncilFlorence, Italy; ^3^Department of Agrifood Production and Environmental Sciences, University of FlorenceFlorence, Italy

**Keywords:** photosynthesis, stomatal conductance, yield, food security, atmospheric CO_2_, elevated CO_2_

## Abstract

Meta-analysis is extensively used to synthesize the results of free air CO_2_ enrichment (FACE) studies to produce an average effect size, which is then used to model likely plant response to rising [CO_2_]. The efficacy of meta-analysis is reliant upon the use of data that characterizes the range of responses to a given factor. Previous meta-analyses of the effect of FACE on plants have not incorporated the potential impact of reporting bias in skewing data. By replicating the methodology of these meta-analytic studies, we demonstrate that meta-analysis of FACE has likely exaggerated the effect size of elevated [CO_2_] on plants by 20 to 40%; having significant implications for predictions of food security and vegetation response to climate change. Incorporation of the impact of reporting bias did not affect the significance or the direction of the [CO_2_] effect.

Meta-analysis is a statistical approach that combines the findings of multiple experimental studies to quantify a population effect (Field and Gillett, 2010; Quintana, 2015). This technique has become increasingly popular to gage plant responses to carbon dioxide (Long et al., 2004; Ainsworth and Long, 2005; Ainsworth and Rogers, 2007), ozone (Feng et al., 2008), nutrient status (Koricheva et al., 1998), herbivory (Hawkes and Sullivan, 2001) and drought (Pinheiro and Chaves, 2011). The synthesis of pools of data from related studies should in theory permit more accurate prediction of the impact of environmental change on plants. Indeed, the results of meta-analytic studies are increasingly used to model plant responses to climate change and inform perspectives on the likely impacts on photosynthesis, carbon sequestration, and food security (Long et al., 2004, 2006; Ainsworth, 2008; Wu et al., 2011). Here, we illustrate how the limitations of this approach are not being critically applied in the plant sciences. One area where meta-analysis has been widely utilized is in the study of plant responses to increased atmospheric carbon dioxide concentration ([CO_2_]) in free air [CO_2_] enrichment (FACE) studies (e.g., Long et al., 2004; Ainsworth and Long, 2005; Ainsworth and Rogers, 2007; Bishop et al., 2014). We use the meta-analysis of FACE experiments as an example of the limitations inherent in this approach that result in an overemphasis of the effect of [CO_2_], and thus distort our understanding of crop responses to [CO_2_]. We acknowledge that growth under FACE has a direct impact upon photosynthesis and growth through CO_2_-fertilization; however, meta-analytic approaches have exaggerated the predicted impact of rising [CO_2_].

Meta-analysis utilizes the effect size of numerous studies to produce an average effect size for a given factor (Field and Gillett, 2010; Quintana, 2015). As such, the meta-analysis is entirely dependent upon the input of studies, and whether those studies represent a true reflection of the treatment effect size. The most highly cited (Long et al., 2004; Ainsworth and Long, 2005; Ainsworth and Rogers, 2007) and recent meta-analytic studies (Bishop et al., 2014; Baig et al., 2015) of plant responses to FACE rely upon data from peer-reviewed studies indexed in the *ISI Web of Science* and/or *Scopus*. However, the possibility of reporting bias influencing the selection of studies is not considered. The issue of reporting bias is widely acknowledged in medicinal science; it is estimated that studies that demonstrate a positive effect are 94% more likely to be submitted (Greenwald, 1975) and then published (Coursol and Wagner, 1986) in leading journals. These journals are most likely to be indexed and their studies included in meta-analyses (**Figure 1**). This skew toward positive studies is driven by publication bias (where journals prefer to publish positive studies), data availability bias (studies with a large effect size are more likely to be written up in comparison to those where the replication is insufficient to demonstrate a significant effect) and reviewer bias (where reviewers favor manuscripts reporting strong treatment effects confirming a prevailing consensus; Dwan et al., 2013). Funnel plots are one of the most common methods to observe possible reporting bias in meta-analysis datasets. Asymmetry in funnel plots is indicative of bias and can be assessed using regression (Egger et al., 1997), rank correlation (Begg and Mazumdar, 1994) and the ‘trim and fill’ method [where estimated ‘missing studies’ are imputed to create a more symmetrical funnel plot (Duval and Tweedie, 2000)].

**FIGURE 1:**
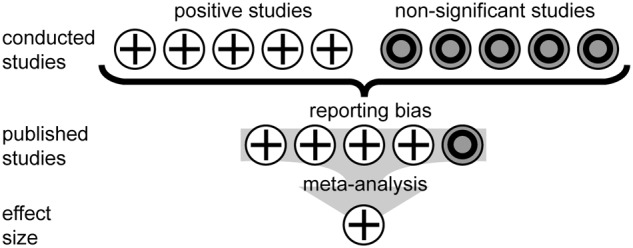
**An illustration of the development of reporting bias in the published literature and its possible effect on the outcome of a meta-analytic study**.

To test for and assess the possible impacts of bias in FACE studies, we followed the methodology of previous meta-analysis of FACE by analysing data from studies indexed in the *ISI Web of Science* (Long et al., 2004; Ainsworth and Long, 2005; Ainsworth and Rogers, 2007; Bishop et al., 2014; Baig et al., 2015). We compiled photosynthesis, stomatal conductance and yield data from 103 studies of C3 herbaceous plants to FACE (a full list of articles and species used in the meta-analysis is given in Supplementary Information). We then performed a random effects meta-analysis using the metafor package (Viechtbauer, 2010) in *R* statistical software following Field and Gillett (2010) and Quintana (2015) (**Figure 2**). Bias in the dataset was assessed using regression (Egger et al., 1997), rank correlation (Begg and Mazumdar, 1994), trim and fill (Duval and Tweedie, 2000) and weighting analysis of the studies (Vevea and Woods, 2005).

**FIGURE 2:**
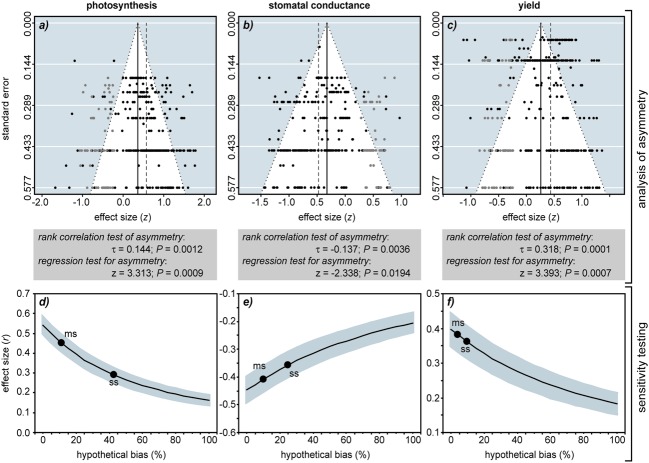
**The impact of reporting bias on the outcome of meta-analysis of the effect of FACE on C3 herbaceous plants.** All articles were peer-reviewed and listed within the *ISI Web of Knowledge*. Funnel plots of photosynthesis (*n* = 265) **(a)**, stomatal conductance (*n* = 243) **(b)** and yield (*n* = 302) **(c)** show the distribution of data. Data from the studies used in the meta-analysis is represented by solid black circles. To balance asymmetry in the funnel plot the trim and fill method (Duval and Tweedie, 2000) uses the existing data to impute estimated ‘missing studies’ that are represented as gray symbols. The black solid vertical line indicates the mean effect size of the meta-analysis after the trim and fill. The dashed vertical line indicates the mean effect size computed by the meta-analysis before the ‘missing studies’ were imputed. The difference between the black solid line and dashed vertical line represents the effect of *reporting bias* on effect size as indicated by the trim and fill method. The gray box below the funnel plot shows the Begg – Mazumdar (Begg and Mazumdar, 1994) rank correlation coefficient using Kendall’s τ and Egger’s regression test (Egger et al., 1997) to assess the probability of publication bias within the datasets. To gage the impact of hypothetical publication bias in the literature on the meta-analysis of photosynthesis **(d)**, stomatal conductance **(e)** and yield **(f)** we included increasing numbers of studies with randomly generated small effect sizes (*r*) of -0.1 to 0.1 (Cohen, 1992). The black solid line indicates the mean effect size for the meta-analysis and the gray shading either side represents 95% confidence intervals. Solid circles indicate the points where moderate (labeled ms) and severe (labeled ss) selection calculated using the model of Vevea and Woods (2005) would occur.

Begg and Mazumdar’s (1994) rank correlation and Egger’s et al. (1997) regression test indicated significant asymmetry in the funnel plots suggestive of reporting bias for all three parameters. The inclusion of estimated missing studies using the trim and fill method (Duval and Tweedie, 2000) resulted in a more balanced spread of the data and also reduced the effect size of FACE on photosynthesis by 43%, stomatal conductance by 32% and yield by 41%. The model of Vevea and Woods (2005) performs a sensitivity analysis, applying weight functions of the effect sizes of studies within a meta-analysis to determine the impact of moderate or severe reporting bias on effect size. Assuming that our dataset has experienced moderate selection, this would indicate that reporting bias has induced 5 to 15% increases in effect size.

It is particularly difficult to quantify the true effect of bias on a meta-analysis (Field and Gillett, 2010; Quintana, 2015). It is possible to survey non-indexed so-called ‘gray’ literature that is not subject to peer-review, directly approach researchers for non-significant unpublished data or submit contrasting ‘sample’ articles or questionnaires to journals to quantify rates of acceptance/rejection. However, all of these methods are time consuming and subject to limitations. We therefore decided to assess the potential impact of bias on meta-analysis of FACE by incorporating an increasing proportion of studies showing small effect sizes (randomly generated *r* values of -0.1 to 0.1: Cohen (1992) and re-running the meta-analyses as a ‘sensitivity test’ of the published data). Assuming that the current published literature is not subject to any bias, photosynthesis (*r* = 0.542), stomatal conductance (*r* = -0.447), and yield (*r* = 0.398) all showed significant effects of elevated [CO_2_], and the significance of this effect remained even at the highest levels of hypothetical reporting bias. A hypothetical publication bias of 30% induced reductions in [CO_2_] effect size of 43.7% in photosynthesis, 27.6% in stomatal conductance and 27.5% in yield. The decline in effect size becomes more apparent at the 80–90% level found in medicinal science (Greenwald, 1975; Coursol and Wagner, 1986). Such reductions in effect size will have critical implications for studies where the output of meta-analyses are used to predict the photosynthetic (Ainsworth and Rogers, 2007) and yield (Long et al., 2006; Bishop et al., 2014; Challinor et al., 2014) responses of plants to rising [CO_2_].

Our analysis is indicative of high levels of bias within published meta-analytic studies of plant responses to FACE that have resulted in over-estimation of the effect size of elevated [CO_2_]. As a result the outputs of these studies should be treated with a degree of caution. We propose that sensitivity testing of meta-analytic studies of plant responses to FACE be undertaken as standard in the future (e.g., Vevea and Woods, 2005), and efforts made to further encourage the publication of studies reporting non-significant outcomes and compilation of non-significant data for researchers wishing to undertake meta-analysis.

## Author Contributions

MH and YH conceived the study. MH and YH collected the data. DK performed statistical analysis. MH drew the figures. MH, YH, and DK wrote the manuscript.

## Conflict of Interest Statement

The authors declare that the research was conducted in the absence of any commercial or financial relationships that could be construed as a potential conflict of interest.
