# Supplementary material for: Has the Impact of Rising CO2 on Plants been Exaggerated by Meta-Analysis of Free Air CO2 Enrichment Studies?
Source: Front Plant Sci. 2016 Aug 3;7:1153. doi: 10.3389/fpls.2016.01153 (PMC4971589; doi:10.3389/fpls.2016.01153)
Supplement: Supplementary file 1 [file Data_Sheet_1.DOCX]

Supplementary information: FACE studies utilised in the meta-analysis of [CO_2_] effect sizes and the potential impact of reporting bias.

| First author | Year | Journal | Volume | Page numbers | Species |
| --- | --- | --- | --- | --- | --- |
| von Caemmerer | 2001 | Aust. J. Plant Physiol. | 28 | 439–450 | Lolium perenne |
| von Caemmerer | 2001 | Aust. J. Plant Physiol. | 28 | 439–450 | Trifolium subterraneum |
| von Caemmerer | 2001 | Aust. J. Plant Physiol. | 28 | 439–450 | Trifolium repens |
| von Caemmerer | 2001 | Aust. J. Plant Physiol. | 28 | 439–450 | Paspalum dilatatum |
| Anderson | 2001 | GCB | 7 | 693-707 | Bromus japonicus |
| Anderson | 2001 | GCB | 7 | 693-707 | Bothriochloa ischaemum |
| Anderson | 2001 | GCB | 7 | 693-707 | Solanum dimidiatum |
| Hebeisen | 1997 | GCB | 3 | 149-160 | Trifolium repens |
| Hebeisen | 1997 | GCB | 3 | 149-160 | Lolium perenne |
| Leakey | 2006 | Plant, Cell and Environment | 29 | 1794-1800 | Glycine max |
| Lee | 2001 | New Phytologist | 150 | 405-418 | Agropyron repens |
| Lee | 2001 | New Phytologist | 150 | 405-418 | Bromus inermis |
| Lee | 2001 | New Phytologist | 150 | 405-418 | Koeleria cristata |
| Lee | 2001 | New Phytologist | 150 | 405-418 | Achillea millefolium |
| Lee | 2001 | New Phytologist | 150 | 405-418 | Anemone cylindrica |
| Lee | 2001 | New Phytologist | 150 | 405-418 | Solidago rigida |
| Maunet | 1994 | Agricultural and Forest Meteorology | 70 | 49-67 | Gossypium hirsutum |
| Miglietta | 1996 | Photosynthesis Research | 3 | 281-290 | Triticum aestivum |
| Nijs | 1997 | Plant, Cell and Environment | 20 | 1041-1050 | Lolium perenne |
| rogers | 2004 | Plant, Cell and Environment | 27 | 449–458 | Glycine max |
| Manderscheid | 2010 | European Journal of Agronomy | 32 | 228–239 | Beta vulgaris |
| Kim | 2003 | Field Crops Research | 83 | 261–270 | Oryza sativa |
| DeMatta | 2016 | Journal of Experimental Botany | 67 | 341–352 | Coffea arabica |
| Jin | 2015 | Annals of Botany | 116 | 975–985 | Pisum sativum |
| Ruhil | 2015 | Protoplasma | 252 | 935–946 | Brassica juncea |
| Bunce | 2014 | Plant Science | 226 | 131–135 | Glycine max Clark day FACE |
| Bunce | 2014 | Plant Science | 226 | 131–135 | Glycine max Clark 24h FACE |
| Bunce | 2014 | Plant Science | 226 | 131–135 | Glycine max Spencer day FACE |
| Bunce | 2014 | Plant Science | 226 | 131–135 | Glycine max Spencer 24h FACE |
| Bunce and Sicher | 2001 | Photosynthetica | 39 | 95-101 | Glycine max |
| Gao | 2015 | Journal of Integrative Agriculture | 14 | 977–983 | Vigna radiata |
| Usui | 2014 | Rice | 7 | 6 | Akitakomachi |
| Usui | 2014 | Rice | 7 | 6 | Kinuhikari |
| Usui | 2014 | Rice | 7 | 6 | Koshihikari |
| Usui | 2014 | Rice | 7 | 6 | Nipponbare |
| Usui | 2014 | Rice | 7 | 6 | Matsuribare |
| Usui | 2014 | Rice | 7 | 6 | HT Mineharuka |
| Usui | 2014 | Rice | 7 | 6 | Toyama |
| Usui | 2014 | Rice | 7 | 6 | Eminokizuna |
| Usui | 2014 | Rice | 7 | 6 | Wa2398 |
| Usui | 2014 | Rice | 7 | 6 | Kanto |
| Usui | 2014 | Rice | 7 | 6 | Kanto |
| Usui | 2014 | Rice | 7 | 6 | Saikai |
| Zhang | 2013 | Photosynthetica | 51 | 593-602 | Triticum aestivum |
| Sharma | 2014 | International Journal of Greenhouse Gas Control | 24 | 139–148 | Taraxacum officinale |
| Sharma | 2014 | International Journal of Greenhouse Gas Control | 24 | 139–148 | Dactylis glomerata |
| Hao | 2013 | Plos One | 8 | e74600 | Isatis indigotica |
| Albert | 2011 | Journal of Experimental Botany | 62 | 4253–4266 | Deschampsia flexuosa |
| Bernacchi | 2005 | Planta | 220 | 434–446 | Glycine max cv. Pana |
| Bernacchi | 2005 | Planta | 220 | 434–446 | Glycine max cv. 93B15 |
| Del Pozo | 2005 | Plant Science | 169 | 908–916 | Triticum aestivum |
| Li | 2013 | Physiologia Plantarum | 148 | 261–272 | Fargesia rufa |
| Bunce | 2012 | Photosynthetica | 50 | 395-400 | Gossypium hirsutum |
| Bunce | 2012 | Photosynthetica | 50 | 395-400 | Triticum aestivum |
| Yoshimoto | 2005 | Agricultural and Forest Meteorology | 133 | 226–246 | Oryza sativa |
| Wall | 2011 | Agriculture, Ecosystems and Environment | 144 | 390–404 | Hordeum vulgare |
| Tausz-Posch | 2013 | Physiologia Plantarum | 148 | 232–245 | Triticum aestivum cv Drysdale |
| Tausz-Posch | 2013 | Physiologia Plantarum | 148 | 232–245 | Triticum aestivum cv Hartog |
| Shimono | 2010 | Plant, Cell and Environment | 33 | 322–331 | Oryza sativa |
| Salazar-Parra | 2011 | Annals of Applied Biology | 161 | 277–292 | Vitis vinifera |
| AESCHLIMANN | 2005 | Plant, Cell and Environment | 28 | 823–833 | Lolium perenne |
| AESCHLIMANN | 2005 | Plant, Cell and Environment | 28 | 823–833 | Trifolium repens |
| Aranjuelo | 2011 | Oecologia | 167 | 339–354 | Larrea tridentata |
| Bishop | 2015 | Plant, Cell and Environment | 38 | 1765–1774 | Glycine max |
| BORJIGIDAI | 2006 | Annals of Botany | 97 | 549–557 | Oryza sativa |
| Burkart | 2009 | Plant Biology | 11 | 109–123 | Beta vulgaris |
| Chen | 2014 | Plant Cell Physiology | 52 | 381–391 | Oryza sativa L. cv. Takanari |
| Butterly | 2016 | Annals of Botany | 117 | 177–185 | Pisum sativum |
| Crous | 2010 | Global Change Biology | 16 | 2076–2088 | Poa pratensis |
| Crous | 2010 | Global Change Biology | 16 | 2076–2088 | Koeleria cristata |
| Crous | 2010 | Global Change Biology | 16 | 2076–2088 | Bromus inermis |
| Crous | 2010 | Global Change Biology | 16 | 2076–2088 | Agropyron repens |
| Crous | 2010 | Global Change Biology | 16 | 2076–2088 | Solidago rigida |
| Crous | 2010 | Global Change Biology | 16 | 2076–2088 | Anemone cylindrica |
| Crous | 2010 | Global Change Biology | 16 | 2076–2088 | Anemone millefolium |
| Ghini | 2015 | Climatic Change | 132 | 307–320 | Coffea arabica catuai |
| Ghini | 2015 | Climatic Change | 132 | 307–320 | Coffea arabica Obata |
| Hao | 2012 | Photosynthetica | 50 | 362-370 | Glycine max cv Zhonghuang 13 |
| Hao | 2012 | Photosynthetica | 50 | 362-370 | Glycine max cv Zhonghuang 35 |
| Hao | 2014 | Agriculture, Ecosystems and Environment | 192 | 80-84 | Glycine max |
| Locke | 2013 | Annals of Botany | 112 | 911–918 | Glycine max |
| Liu | 2009 | Water Air Soil Pollution | 212 | 387–394 | Eichhornia crassipe |
| Manderscheid | 2009 | Field Crops Research | 110 | 185–196 | Hordeum vulgare |
| Sanz-Sáez | 2015 | Plant, Cell and Environment | 38 | 2589–2602 | Glycine max Merr |
| Shimono | 2009 | Journal of Experimental Botany | 60 | 523–532 | Oryza sativa |
| Tausz-Posch | 2012 | Field Crops Research | 133 | 160–166 | Triticum aestivum Drysdale |
| Yong | 2007 | Photosynthetica | 45 | 85-91 | Oryza sativa |
| Zhu | 2014 | Journal of Experimental Botany | 65 | 6049–6056 | Oryza sativa S63 |
| Zhu | 2014 | Journal of Experimental Botany | 65 | 6049–6056 | Oryza sativa W14 |
| Zhu | 2015 | Scientific Report | 5 | 12719 | Oryza sativa |
| Morgan | 2005 | Global Change Biology | 11 | 1856–1865 | Glycine max (L.) Merr |
| Yang | 2007 | Field Crops Research | 102 | 128–140 | Oryza sativa |
| Högy | 2009 | Plant Biology | 11 | 60–69 | Triticum aestivum L. cv. TRISO |
| Bloom | 2010 | Sience | 328 | 899-903 | Triticum aestivum |
| Ellsworth | 2004 | Global Change Biology | 10 | 2121–2138 | Achillea millefolium |
| Ellsworth | 2004 | Global Change Biology | 10 | 2121–2138 | Agropyron repens |
| Ellsworth | 2004 | Global Change Biology | 10 | 2121–2138 | Anemone cylindrica |
| Ellsworth | 2004 | Global Change Biology | 10 | 2121–2138 | Bromus inermis |
| Ellsworth | 2004 | Global Change Biology | 10 | 2121–2138 | Lupinus perennis |
| Ellsworth | 2004 | Global Change Biology | 10 | 2121–2138 | Poa pratensis |
| Ellsworth | 2004 | Global Change Biology | 10 | 2121–2138 | Solidago rigida |
| Ellsworth | 2004 | Global Change Biology | 10 | 2121–2138 | Oenothera primiveris |
| Chen | 2005 | Plant Cell Physiology | 46 | 1036–1045 | Oryza sativa cv. Japonica 9915 |
| Hasegawa | 2013 | Functional Plant Biology | 40 | 148-159 | AkitakomachiS-2007 |
| Hasegawa | 2013 | Functional Plant Biology | 40 | 148-159 | S-2008 |
| Hasegawa | 2013 | Functional Plant Biology | 40 | 148-159 | T-2010 |
| Hasegawa | 2013 | Functional Plant Biology | 40 | 148-159 | Akita 63 S-2007 |
| Hasegawa | 2013 | Functional Plant Biology | 40 | 148-159 | S-2008 |
| Hasegawa | 2013 | Functional Plant Biology | 40 | 148-159 | T-2010 |
| Hasegawa | 2013 | Functional Plant Biology | 40 | 148-159 | Koshihikari S-2007 |
| Hasegawa | 2013 | Functional Plant Biology | 40 | 148-159 | S-2008 |
| Hasegawa | 2013 | Functional Plant Biology | 40 | 148-159 | T-2010 |
| Hasegawa | 2013 | Functional Plant Biology | 40 | 148-159 | Takanari S-2007 |
| Hasegawa | 2013 | Functional Plant Biology | 40 | 148-159 | S-2008 |
| Hasegawa | 2013 | Functional Plant Biology | 40 | 148-159 | T-2010 |
| Chen | 2008 | 14th International Congress on Photosynthesis |  | 1367–1370 | Oryza sativa |
| Adam | 2000 | Photosynthesis Research | 66 | 65-77 | Triticum aestivum |
| Adam | 2000 | Photosynthesis Research | 66 | 65-77 | Triticum aestivum |
| Ainsworth | 2003 | Plant, Cell and Environment | 26 | 705-714 | Lolium perenne |
| Ainsworth | 2003 | Journal of Experimental Botany | 54 | 2769-2774 | Trifolium repens |
| Ainsworth | 2004 | Agricultural and Forest Meteorology | 122 | 85-94 | Glycine max |
| Bindi | 2001 | European Journal of Agronomy | 14 | 145-155 | Vitis vinifera |
| Brooks | 2000 | Photosynthesis Research | 66 | 97-108 | Tritucum aestivum |
| Bryant | 1998 | Plant, Cell and Environment | 21 | 159-168 | Anthyllis vulneraria |
| Bryant | 1998 | Plant, Cell and Environment | 21 | 159-168 | Sanguisorba minor |
| Bryant | 1998 | Plant, Cell and Environment | 21 | 159-168 | Bromopsis erecta |
| Daepp | 2000 | GCB | 6 | 805-816 | Lolium perenne |
| Derner | 2003 | GCB | 9 | 452-460 | Gossypium hirsutum |
| Edwards | 2001 | New Phytologist | 150 | 359-369 | Anthoxanthum odoratum |
| Edwards | 2001 | New Phytologist | 150 | 359-369 | Lorium perenne |
| Ewert | 2002 | Agriculture, Ecosystems and Environment | 93 | 249-266 | Triticum aestivum L. |
| Garcia | 1998 | Plant, Cell and Environment | 21 | 659-669 | Triticum aestivum L. |
| Hileman | 1994 | Agricultural and Forest Meteorology | 70 | 189-207 | Gossypium hirsutum |
| Huxman and Smith | 2001 | Oecologia | 128 | 193-201 | Bromus mafritensis |
| Huxman and Smith | 2001 | Oecologia | 128 | 193-201 | Eriogonum infiatum |
| Huxman and Smith | 2001 | Oecologia | 128 | 193-201 | Bromus mafritensis |
| Huxman and Smith | 2001 | Oecologia | 128 | 193-201 | Eriogonum infiatum |
| Idso | 1994 | Agricultural and Forest Meteorology | 70 | 183-188 | Gossypium hirsutum |
| Isopp | 2000 | Plant, Cell and Environment | 23 | 597-607 | Lolium perenne |
| Kim | 2001 | New Phytologist | 150 | 223-229 | Oryza sativa |
| Kim | 2001 | New Phytologist | 150 | 223-229 | Oryza sativa |
| Kimball | 1995 | GCB | 1 | 429-442 | Tritucum aestivum, WW, 16 Mar 1993 |
| Luscher | 1998 | Oecologia | 113 | 37-45 | Arrhenatherum elatius,1993 summer |
| Luscher | 1998 | Oecologia | 113 | 37-45 | Dactylis glomerata, 1993 summer |
| Luscher | 1998 | Oecologia | 113 | 37-45 | Festuca pratensis, 1993 summer |
| Luscher | 1998 | Oecologia | 113 | 37-45 | Holcus lanatus, 1993 summer |
| Luscher | 1998 | Oecologia | 113 | 37-45 | Lotium multiflorum, 1993 summer |
| Luscher | 1998 | Oecologia | 113 | 37-45 | Lotium perenne, 1993 summer |
| Luscher | 1998 | Oecologia | 113 | 37-45 | Trisetum flavescens, 1993 summer |
| Luscher | 1998 | Oecologia | 113 | 37-45 | Rumex acetosa, 1993 summmer |
| Luscher | 1998 | Oecologia | 113 | 37-45 | Rumex obtusifolius, 1993 summer |
| Luscher | 1998 | Oecologia | 113 | 37-45 | Ranunculus friesianus, 1993 summer |
| Luscher | 1998 | Oecologia | 113 | 37-45 | Trifolium pratense, 1993 summer |
| Luscher | 1998 | Oecologia | 113 | 37-45 | Trofolium repens, 1993 summer |
| Luscher | 1998 | Oecologia | 113 | 37-45 | Arrhenatherum elatius,1994 spring |
| Luscher | 1998 | Oecologia | 113 | 37-45 | Dactylis glomerata, 1994 spring |
| Luscher | 1998 | Oecologia | 113 | 37-45 | Festuca pratensis, 1994 spring |
| Luscher | 1998 | Oecologia | 113 | 37-45 | Holcus lanatus, 1994 spring |
| Luscher | 1998 | Oecologia | 113 | 37-45 | Lotium multiflorum, 1994 spring |
| Luscher | 1998 | Oecologia | 113 | 37-45 | Lotium perenne, 1994 spring |
| Luscher | 1998 | Oecologia | 113 | 37-45 | Trisetum flavescens, 1994 spring |
| Luscher | 1998 | Oecologia | 113 | 37-45 | Rumex acetosa, 1994 spring |
| Luscher | 1998 | Oecologia | 113 | 37-45 | Rumex obtusifolius, 1994 spring |
| Luscher | 1998 | Oecologia | 113 | 37-45 | Ranunculus friesianus, 1994 spring |
| Luscher | 1998 | Oecologia | 113 | 37-45 | Trifolium pratense, 1994 spring |
| Luscher | 1998 | Oecologia | 113 | 37-45 | Trofolium repens, 1994 spring |
| Luscher | 1998 | Oecologia | 113 | 37-45 | Arrhenatherum elatius,1994 summer |
| Luscher | 1998 | Oecologia | 113 | 37-45 | Dactylis glomerata, 1994 summer |
| Luscher | 1998 | Oecologia | 113 | 37-45 | Festuca pratensis, 1994 summer |
| Luscher | 1998 | Oecologia | 113 | 37-45 | Holcus lanatus, 1994 summer |
| Luscher | 1998 | Oecologia | 113 | 37-45 | Lotium multiflorum, 1994 summer |
| Luscher | 1998 | Oecologia | 113 | 37-45 | Lotium perenne, 1994 summer |
| Luscher | 1998 | Oecologia | 113 | 37-45 | Trisetum flavescens, 1994 summer |
| Luscher | 1998 | Oecologia | 113 | 37-45 | Rumex acetosa, 1994 summer |
| Luscher | 1998 | Oecologia | 113 | 37-45 | Rumex obtusifolius, 1994 summer |
| Luscher | 1998 | Oecologia | 113 | 37-45 | Ranunculus friesianus, 1994 summer |
| Luscher | 1998 | Oecologia | 113 | 37-45 | Trifolium pratense, 1994 summer |
| Luscher | 1998 | Oecologia | 113 | 37-45 | Trofolium repens, 1994 summer |
| Luscher | 1998 | Oecologia | 113 | 37-45 | Arrhenatherum elatius,1995 spring |
| Luscher | 1998 | Oecologia | 113 | 37-45 | Dactylis glomerata, 1995 spring |
| Luscher | 1998 | Oecologia | 113 | 37-45 | Festuca pratensis, 1995 spring |
| Luscher | 1998 | Oecologia | 113 | 37-45 | Holcus lanatus, 1995 spring |
| Luscher | 1998 | Oecologia | 113 | 37-45 | Lotium multiflorum, 1995 spring |
| Luscher | 1998 | Oecologia | 113 | 37-45 | Lotium perenne, 1995 spring |
| Luscher | 1998 | Oecologia | 113 | 37-45 | Trisetum flavescens, 1995 spring |
| Luscher | 1998 | Oecologia | 113 | 37-45 | Rumex acetosa, 1995 spring |
| Luscher | 1998 | Oecologia | 113 | 37-45 | Rumex obtusifolius, 1995 spring |
| Luscher | 1998 | Oecologia | 113 | 37-45 | Ranunculus friesianus, 1995 spring |
| Luscher | 1998 | Oecologia | 113 | 37-45 | Trifolium pratense, 1995 spring |
| Luscher | 1998 | Oecologia | 113 | 37-45 | Trofolium repens, 1995 spring |
| Luscher | 1998 | Oecologia | 113 | 37-45 | Arrhenatherum elatius,1995 summer |
| Luscher | 1998 | Oecologia | 113 | 37-45 | Dactylis glomerata, 1995 summer |
| Luscher | 1998 | Oecologia | 113 | 37-45 | Festuca pratensis, 1995 summer |
| Luscher | 1998 | Oecologia | 113 | 37-45 | Holcus lanatus,1995 summer |
| Luscher | 1998 | Oecologia | 113 | 37-45 | Lotium multiflorum, 1995 summer |
| Luscher | 1998 | Oecologia | 113 | 37-45 | Lotium perenne, 1995 summer |
| Luscher | 1998 | Oecologia | 113 | 37-45 | Trisetum flavescens, 1995 summer |
| Luscher | 1998 | Oecologia | 113 | 37-45 | Rumex acetosa, 1995 summer |
| Luscher | 1998 | Oecologia | 113 | 37-45 | Rumex obtusifolius, 1995 summer |
| Luscher | 1998 | Oecologia | 113 | 37-45 | Ranunculus friesianus, 1995 summer |
| Luscher | 1998 | Oecologia | 113 | 37-45 | Trifolium pratense, 1995 summer |
| Luscher | 1998 | Oecologia | 113 | 37-45 | Trofolium repens, 1995 summer |
| Haworth | 2016 | Functional Plant Biology | 43 | 26-39 | Arrhenatherum elatius |
| Haworth | 2016 | Functional Plant Biology | 43 | 26-39 | Geranium pratense |
| Haworth | 2016 | Functional Plant Biology | 43 | 26-39 | Holcus lanatus |
| Haworth | 2016 | Functional Plant Biology | 43 | 26-39 | Plantago lanceolata |
| Haworth | 2016 | Functional Plant Biology | 43 | 26-39 | Rumex acetosa |
| Haworth | 2016 | Functional Plant Biology | 43 | 26-39 | Sanguisorba officinalis |
| Manunta | 2002 | Int J Biometeorol | 46 | 9-21 | Triticum aestivum, optimal water |
| Mauney | 1994 | Agricultural and Forest Meteorology | 70 | 49-67 | Gossypium hirsutum, WW, 1990 |
| Miglietta | 1998 | GCB | 4 | 163-172 | Solanum tuberosum |
| Norton | 1999 | Functional Ecology | 13 | 38-44 | Lolium perenne |
| Norton | 1999 | Functional Ecology | 13 | 38-44 | Plantago lanceolata |
| Norton | 1999 | Functional Ecology | 13 | 38-44 | Phleum pratense |
| Norton | 1999 | Functional Ecology | 13 | 38-44 | Poa trivialis |
| Nowak | 2001 | New Phytologist | 150 | 449-458 | Achnatherum hymenoldes |
| Osborne | 1998 | Plant Physiology | 117 | 1037-1045 | Triticum aestivum |
| Reich | 2001 | Nature | 410 | 809-812 | Achilea |
| Reich | 2001 | Nature | 410 | 809-812 | Poa |
| Reich | 2001 | Nature | 410 | 809-812 | Lupinus |
| Reich | 2001 | Nature | 410 | 809-812 | Bromus |
| Reich | 2001 | New Phytologist | 150 | 435-448 | Agropyron repens |
| Reich | 2001 | New Phytologist | 150 | 435-448 | Bormus inermis |
| Reich | 2001 | New Phytologist | 150 | 435-448 | Koeleria cristata |
| Reich | 2001 | New Phytologist | 150 | 435-448 | Poa pratensis |
| Reich | 2001 | New Phytologist | 150 | 435-448 | Achillea millefolium |
| Reich | 2001 | New Phytologist | 150 | 435-448 | Anemone cylindrica |
| Reich | 2001 | New Phytologist | 150 | 435-448 | Asclepias tuberosa |
| Reich | 2001 | New Phytologist | 150 | 435-448 | Solidago rigida |
| Reich | 2001 | New Phytologist | 150 | 435-448 | Amorpha canescens |
| Reich | 2001 | New Phytologist | 150 | 435-448 | Lespedeza capitata |
| Reich | 2001 | New Phytologist | 150 | 435-448 | Lupinus perennis |
| Reich | 2001 | New Phytologist | 150 | 435-448 | Petalostemum villosum |
| Rogers | 1998 | Plant Physiology | 118 | 683-689 | Lolium perenne |
| Rogers | 2004 | Plant, Cell and Environment | 27 | 449-458 | Glycine max |
| Seneweera | 2002 | Functional Plant Biology | 29 | 945-953 | Oryza sativa |
| Shaw | 2001 | Science | 298 | 1987-1990 | Grassland |
| Wall | 2000 | Photosynthesis Research | 66 | 79-95 | Triticum aestivum |
| Warwick | 1998 | GCB | 4 | 375-385 | Sanguisorbe minor |
| Warwick | 1998 | GCB | 4 | 375-385 | Lotus corniculatus |
| Warwick | 1998 | GCB | 4 | 375-385 | Anthyllis vulneraria |
| Warwick | 1998 | GCB | 4 | 375-385 | Plantago media |
| Batthacarya | 1994 | Agricultural and Forest Meteorology | 70 | 171-182 | Gossypium hirsutum |
| Luscher | 2000 | GCB | 6 | 655-662 | Medicago sativa |
| Pearson | 1995 | Plant, Cell and Environment | 18 | 837-843 | Rumex obtusifolius |
| Suter | 2001 | Crop Science | 41 | 810-817 | Lolium perenne |
| Hileman | 1992 | Critical Reviews in Plant Sciences | 11 | 227-231 | Gossypium hirsutum |
| Mauney | 1992 | Critical Reviews in Plant Sciences | 11 | 213-222 | Gossypium hirsutum |
| Asseng | 2004 | Field Crops Research | 85 | 85-102 | Triticum aestivum |
| Grossman-Clarke | 1999 | Agricultural and Forest Meteorology | 93 | 95-109 | Triticum aestivum |
